# Benign Biliary Strictures: A Systematic Review on Endoscopic Treatment Options

**DOI:** 10.3390/diagnostics10040221

**Published:** 2020-04-15

**Authors:** May Y.W. Wong, Payal Saxena, Arthur J. Kaffes

**Affiliations:** 1 Interventional Endoscopy, Chris O Brien Lifehouse, Missenden Road, Camperdown 2050, Australia; wong.may.yw@gmail.com (M.Y.W.W.); psaxena1@jhmi.edu (P.S.); 2 AW Morrow Gastroenterology and Liver Centre, Royal Prince Alfred Hospital, Missenden Road, Camperdown 2050, Australia; 3 Department of Medicine, University of Sydney, Camperdown 2050, Australia

**Keywords:** benign biliary strictures, self-expandable metal stents

## Abstract

Benign biliary strictures can be difficult to manage. Untreated biliary strictures can lead to complications, such as chronic cholestasis, jaundice, recurrent sepsis, and secondary biliary cirrhosis, which can have severe ramifications. The management landscape is constantly evolving, with the development of modifiable self-expandable metal stents and biodegradable stents. This review critically appraises current endoscopic treatment strategies, in particular focusing on the shortfalls, such as stent migration and stricture recurrence. It also proposes a treatment algorithm based on aetiologias and the location of the strictures.

## 1. Introduction

Benign biliary strictures (BBSs) are challenging to manage. Most benign strictures are related to surgical procedures, following bile duct injury after liver transplantation and laparoscopic cholecystectomy, with reported incidences varying from 3% to 13% [1] and 0.2% to 0.7% [2,3], respectively. BBSs can also be related to inflammatory conditions or from other etiologies, such as chronic pancreatitis (CP).

Not all BBSs are the same and they can have varying features depending on the etiology. For example, in CP, BBSs are long and involve the intrapancreatic portion of the common bile duct (CBD). In liver transplantation, BBSs are generally short and narrow. In post-cholecystectomy biliary strictures, BBSs are usually near the main hilar confluence. The approach to the management of BBSs is diverse and includes endoscopic, surgical, and interventional radiological options, all of which can be associated with complications, which can be life threatening in nature, including sepsis, pancreatitis, hemorrhage, and perforation. 

Endoscopic therapy is considered as first-line management secondary to its superior safety profile, efficacy, and less-invasive nature in contrast to surgical or percutaneous techniques. However, there is a possibility of stricture recurrence, and with stent insertion, there is a risk of stent occlusion and migration. The endoscopic technique for BBSs is continuously advancing and we have previously proposed an endoscopic classification to assist with treatment therapies dependent on the location and etiology of biliary stricture [4].

Plastic stents have been the mainstay of treatment in conjunction with endoscopic biliary dilatation. However, this approach can be time and labor intensive, demanding multiple interventional procedures to upsize and exchange stents. This also increases the chance for complications, such as cholangitis and bleeding, and places large demands on healthcare resources. There has therefore been interest in exploring the use of self-expandable metal stents (SEMSs). This technique requires reduced procedures and may also provide faster stricture resolution. However, limitations include a higher risk of migration. The European Society of Gastroenterology Endoscopy (ESGE) guidelines reinforce that multiple plastic stents (MPSs) and SEMSs are the standard of care currently for the treatment of BBSs (strong recommendation, moderate-quality evidence) [5]. Newer fully covered and intraductal SEMSs have antimigration properties to reduce the risk of stent migration [6].

It is important to note that many studies are retrospective with high variability, and as such, many recommendations made by ESGE are based on low- or moderate-quality evidence, particularly those pertaining to SEMSs, as well as the duration of stenting. 

## 2. Plastic Stents

The original standard of care was to place size 10 French plastic stents across strictures. The stents were then slowly upsized and exchanged every 3 months accompanied by dilatation for up to one year [7]. It is important to note that a single plastic stent is now rendered obsolete following a meta-analysis including over 1000 patients that demonstrated that MPS led to greater clinical success and fewer adverse events compared to a single stent [8]. However, there is immense variability with the frequency and duration with this protocol. Newer protocols place maximal numbers of stents across the BBS at the index endoscopic retrograde cholangio-pancreatography (ERCP) following dilatation, which is termed an aggressive MPS placement. It is thought that this can maintain a stretched duct diameter with superior resolution results [9]. Other protocols remove every stent placed previously and re-insert a maximum number of large-diameter plastic stents, inducing progressive stretching of the fibrotic biliary stricture. A retrospective study demonstrated that treatment success was proportional to the number of stents used in total and per ERCP [10] (6 to 9 size 10 French biliary stents used per ERCP). In total, 94% achieved stricture resolution, and with a 11-month median follow up, 3% had recurrence. It is thought that this approach can allow an extended time between ERCPs and also limit cholangitis complications by providing ample biliary flow. Where multiple stents are used, the interval for exchange is not thought to affect the incidence of stent occlusion, and it is not significantly different for stents exchanged within half a year compared to stents exchanged after one year, with resolution rates [11]. ESGE guidelines recommend insertion of the maximum number of stents possible every 3–4 months for a total duration of 12 months (weak recommendation, low-quality evidence) [5].

## 3. Metal Stents

SEMSs address some of the limitations of plastic stents; they have a larger diameter as well as a greater expansion force. SEMSs have wider diameters (10 vs. 3.3 mm, respectively) when compared to plastic stents and also have technical advantages as they are easier to insert and less time arduous than placing MPS [12]. The greater radial force applies a constant and the greatest dilation of the BBS during the initial ERCP as well as allowing for greater intervals between stent exchanges. In addition, the delivery sheath of SEMS is narrower and therefore no prior dilatation is necessary.

The SEMSs used in BBS are all fully covered to prevent tissue ingrowth and stent epithelization into the duct wall, making removal possible. Most are made from a polytetrafluoroethylene layer and have drawstrings situated at the top margin of the stent to facilitate subsequent removal. The stents described from here on are all fully covered SEMSs. 

In general, stricture resolution and remodeling of the bile ducts appears to be quicker with SEMS compared to MPS, requiring less than the total 12 months of stent exchange duration required with plastic stents. This shortened period is thought to reduce the rate of stent-related complications in particular with stent removal without compromising stricture resolution.

It is imperative to select the right stent for the right indication and location. The major determining features are based on the stricture length, location, and etiology. SEMSs are best suited to CP-related strictures and some cases post surgery. The use of SEMSs in strictures at the hilar confluence is not recommended due to the risk of impaction as well as occlusion of the intrahepatic branches from the opposite lobe, which can lead to sepsis [5]. If the stent covers the cystic duct, then cholecystitis can occur if the patient has a gallbladder in situ [12]. Furthermore, SEMSs do not perform well for cholecystectomy and anastomotic strictures due to the excessive migration and recurrence of strictures.

Unlike plastic stents, there are less vigorous studies of the dilatation protocols for SEMS in BBS. Furthermore, the optimal SEMS indwelling duration is unknown. Current practice with SEMSs placed for anastomotic strictures employ a 3-month placement; however, longer treatment times may be more effective for stricture resolution, as seen in instances for CP where stents are placed for up to a year [13]. 

There are concerns that an extended SEMS indwelling time may raise the risk of cholangitis from stent occlusion or migration. There are also risks with stent embedment from the mucosal hyperplasia occurring at the tips of the stent [13]. One large study demonstrated no failures to remove an SEMS at the first follow-up ERCP. This may be related to the study’s enrolment criteria and scheduled 6-month removal time [14]. ESGE guidelines recommend insertion of an 8–10-mm-diameter SEMS for a dwell stenting period of 6 months (weak recommendation, low-quality evidence) [5]. Other limitations of SEMSs include the risk of duct injury, such as small extrahepatic ducts after liver transplantation. 

## 4. Metal vs. Plastic Stents

Several meta-analyses have demonstrated that SEMSs were similar to MPS for BBS resolution whilst requiring fewer ERCP procedures [15,16]. In one meta-analysis, surgery yielded the highest long-term stricture resolution rate (84%), but the difference with other treatment modalities was not significant [17]. However, all meta-analyses used studies with small numbers. In addition, there was great heterogeneity within the studies themselves, with many not detailing the types of prosthesis or design for the stents [13] used nor the exchange frequency. The protocols varied greatly within studies, with I^2^ being quoted from 57% [18] to 96% [17] without elaboration in the discussion. Meta-analyses may not be the best modality to answer the debate between metal vs. plastic stents.

### 4.1. Treatment According to Type of Stricture

Developing a gold standard endoscopic therapy for BBS is limited by the fact that there is no accepted classification for BBS. Whilst the Bismuth classification for malignant biliary strictures can assist with clinical decisions, the equivalent classification for BBS has limited applicability to endoscopic interventions [19]. We refer to our classification reported in 2015 [4], where we divide BBS into four types of strictures, which has corresponding treatment modalities (Table 1).

### 4.2. Type 1 Strictures

Type 1 strictures are located at the distal CBD and occur in conditions, such as in CP. Here, biliary strictures are due to extrinsic compression from fibrotic pancreatic tissue on the bile ducts. It is important that cancer is ruled out before placing stents for 10–12 months at a time. SEMSs have been studied extensively; a systematic review found CP-related BBS attained increased resolution at one year than MPSs (77% vs. 33%, respectively) [20]. Long-term outcomes are also positive; a prospective study found that more than 60% of patients with symptomatic BBS from CP [21] remained asymptomatic and stent free for up to 5 years after the placement of a single SEMS with an acceptable safety profile. 

Overall, outcomes are less positive for CP than for anastomotic strictures after liver transplantation. This is related to the fact that BBSss in CP are generally longer and more complex. Furthermore, there is fibrosis and calcification of the pancreatic parenchyma related to chronic inflammatory changes. ESGE guidelines recommend temporary insertion of MPSs or of an SEMS for treatment of BBS related to CP (weak recommendation, moderate-quality evidence) [5].

### 4.3. Type 2 Strictures

Type 2 strictures occur in the upper CBD or common hepatic duct, such as a liver transplant anastomotic stricture or post-cholecystectomy strictures. Placement of an incremental number of plastic stents exchanged every 3 months over one year is the standard of care for these strictures [9]. 

Post-cholecystectomy BBSs are generally short and occur frequently at the upper third of the common hepatic duct or hilar confluence. As a result, SEMSs are less attractive because of the risk of impaction of the upper edge into the hilar roof as well as the risk of stent migration secondary to unstable positioning of the deployed stent. Furthermore, they have not been well studied and are under-represented in multi-center trials [13,14]. ESGE suggests temporary insertion of MPS for the treatment of BBS, complicating cholecystectomy; an SEMS can be an alternative for strictures located >2 cm from the main hepatic confluence (weak recommendation, moderate-quality evidence) [5].

Post-liver transplant, strictures can be anastomotic biliary strictures (ABSs) or non-anastomotic biliary strictures. For anastomotic biliary strictures, MPS is the gold standard [22]. Meta-analyses [22,23] found SEMS and MPS had equal ABS resolution and recurrence (odds ratio 1.05, 95% confidence interval 0.43–2.56), although there was a trend towards a higher recurrence rate in SEMS. This greater stricture recurrence rate was no longer significant when trials with a shorter stent indwelling time were excluded [22], with benefits for 6 months compared to 3 months [24,25]. ESGE guidelines recommend temporary insertion of MPS for the treatment of benign ABS following orthotopic liver transplantation pending further evidence about SEMS (weak recommendation, moderate-quality evidence) [5].

Non-anastomotic biliary strictures are strictures that occur more than 5 mm proximally to the anastomosis. These are characterized by multiple extrahepatic and/or intrahepatic ABS with recurrent sludge or stone formation. They are more refractory to endoscopic therapy than ABS, requiring greater numbers of stricture dilatation, longer periods of stenting, and have greater rates of stricture recurrence. 

The issues with stent migration and the possibility of stent-induced biliary strictures is ever present, particularly if the diameter of the stent is not adapted to the caliber of the CBD. Various strategies have been used address migration issues. These include the insertion of a double-pigtail plastic stent within an SEMS as an anchor [26]. This resulted in a high stricture resolution rate (89.5%) with a low stricture recurrence rate (17.6%), without any reported SEMS migrations. Other strategies have been derived from esophageal SEMS, where anchoring fins and flaps have been used to modify the typical cylindrical appearance of biliary SEMS [27,28,29]. The CBD is spindle shaped and narrows towards the papilla. As such, anatomy-conforming stents in the shape of single or double cones also have zero or lower migration rates [30,31]. A meta-analysis [18] of post-operative BBS found modified SEMSs reduced stricture migration and the recurrence rates compared with traditional SEMSs. The effect size of stent migration in the modified SEMS group was only 0.03 and was similar to that in the MPS group (0.04), which was significantly lower than the traditional SEMS group (0.25). Hence modified SEMSs may be more favorable than traditional SEMSs, with less stent migration, but future randomized controlled trials (RCTs) are needed.

Results for newer intraductal SEMSs, stents that are located completely within the bile duct [32], have been promising for strictures following liver transplantation. These designs allow for intraductal placement without bridging the ampulla and creation of bilio-enteric anastomosis (Kaffes stent, Niti-S SEMS, Taewoong Medical, S Korea) and have been shown to lower migration rates significantly to 0%–3% [6,32,33] in contrast to greater than 30% for traditional stents.

Newer endoscopic techniques, such as stenting above the papilla, “inside stent”, have been studied to prolong stent patency, especially in patients post-stent patency. This works as a core stent to prevent kinking of the bile duct at the end of the SEMS as well as preventing duodenobiliary reflux [34]. 

### 4.4. Type 3 Strictures

Type 3 strictures are intrahepatic, including the hilum, such as in primary sclerosing cholangitis (PSC). Like in CP, it is important to exclude malignancy where appropriate prior to endoscopic therapy. In the first randomized trial of patients with PSC and a dominant stricture, short-term stents were not superior to balloon dilatation and in fact may be harmful. The study was terminated early due to a significantly higher occurrence of treatment-related significant adverse events for short term stenting, including cholangitis, post-ERCP pancreatitis, and cholecystitis. As a result, balloon dilatation should be the initial treatment of choice for dominant strictures in patients with PSC as it can result in a decrease in cholestasis in the majority of patients at 3 months [35]. Following this study, there are no recommendations for the use of SEMS in PSC. In some patients, to achieve maximal clinical benefit, repeated dilation sessions may be necessary.

### 4.5. Type 4

Type 4 are surgical anastomoses, such as hepaticojejunostomy, which can be challenging for management due to an altered anatomy. The approach has traditionally been via percutaneous transhepatic biliary drainage (PTBD), but if a center has expertise and availability, then double balloon enteroscopy (DBE)-assisted ERCP is an attractive option. A retrospective cohort (102 patients) with repeated DBE-assisted ERCP and balloon dilatation and insertion of a plastic stent if required demonstrated a high stricture resolution rate (77%). This was a vigorous and time-intensive protocol necessitating monthly visits. There was also a high stricture recurrence in 42%, with a median time to recurrence of 3.4 months, and cholangitis in 10% of cases [36]. A meta-analysis of 15 studies showed overall success rates of 81% for DBE to reach the biliary anastomosis of papilla and 62% for achieving successful biliary intervention [37].

### 4.6. Future

Current evidence for all described interventions shows a high stricture resolution rate. However, the main limiting factor is stricture recurrence. There is ongoing research into strategies best to manage SEMS migration, which would allow for stents to remain longer in situ. We have discussed the properties of newer stents with antimigration strategies. There is also interest regarding biodegradable stents; stent removal is not required due to spontaneous stent disintegration. These items can be custom-made in the desired size and length depending on the BBS. However, there is concern regarding the gradual reduction in expansive radial force as the stent degrades as well as initiating a potential inflammatory foreign body reaction. In a multicenter study, 107 patients with refractory BBS [38] and biodegradable stents demonstrated stricture recurrence in 18% of patients. Future directions could look at whether drug delivery, such as via drug-eluting stents, could also be considered to prevent stent ingrowth. 

## 5. Conclusions

The current endoscopic management algorithm of BBS has evolved in the last 20 years. There is constant research and innovation to reflect the unique etiologies and site of strictures. Endoscopic techniques, protocols, and materials are constantly changing. Currently, the stenting protocol with multiple plastic stents is the standard of care for post-surgery BBS. SEMSs are an excellent alternative in many situations, such as chronic pancreatitis. The evolution of biodegradable stents could provide logistical benefits. More studies are required for stronger recommendations to be made by guidelines. 

## Figures and Tables

**Table 1:** Treatment modality according to stricture location and etiology.

Type	Site	Common Etiology	Endoscopic Treatment
1	Distal CBD	Chronic pancreatitis Papillary stenosis	Multiple plastic stent SEMS
2	Mid extra hepatic duct (>1 cm from hilum)	Post surgery (CCY, AS, OLT) PSC	MPS Intraductal SEMS
3	Hilar and intra-hepatic	PSC, autoimmune, ischemic	Balloon dilatation, plastic stenting (single or multiple)
4	Surgical bilio-enteric anastomotic	Post surgical	Balloon dilatation

AS anastomotic stricture, CBD common bile duct, CCY cholecystectomy, MPS multiple plastic stents, OLT liver transplantation, PSC primary sclerosing cholangitis, SEMS self-expandable metal stent [4].
